# Boutonnière Deformity Following Volar Proximal Interphalangeal Joint Dislocation

**Published:** 2016-06-06

**Authors:** Aditya Sood, Vasanth S. Kotamarti, Mark S. Granick

**Affiliations:** Division of Plastic and Reconstructive Surgery, Rutgers New Jersey Medical School, Newark

**Keywords:** finger dislocation, volar dislocation, interphalangeal joint, *boutonnière* deformity, late deformity

## DESCRIPTION

**Figure 1:**
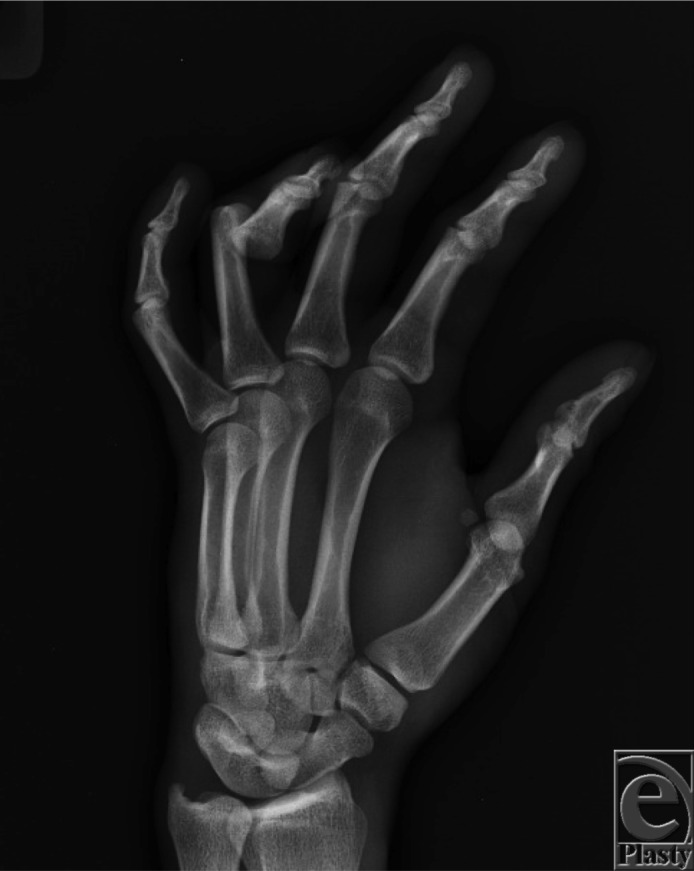
Radiograph from initial presentation demonstrating volar proximal interphalangeal joint dislocation of the fourth digit.

**Figure 2:**
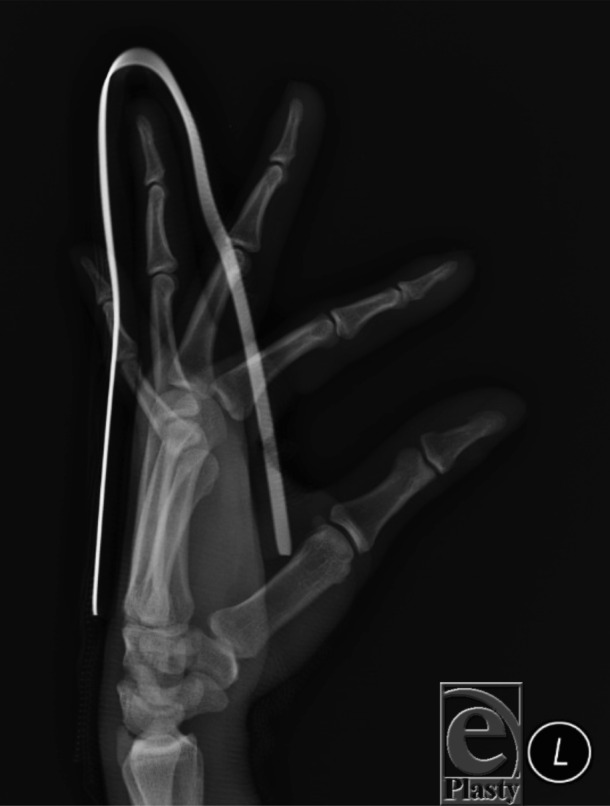
Postreduction radiograph and splinting of the affected finger in extension.

**Figure 3:**
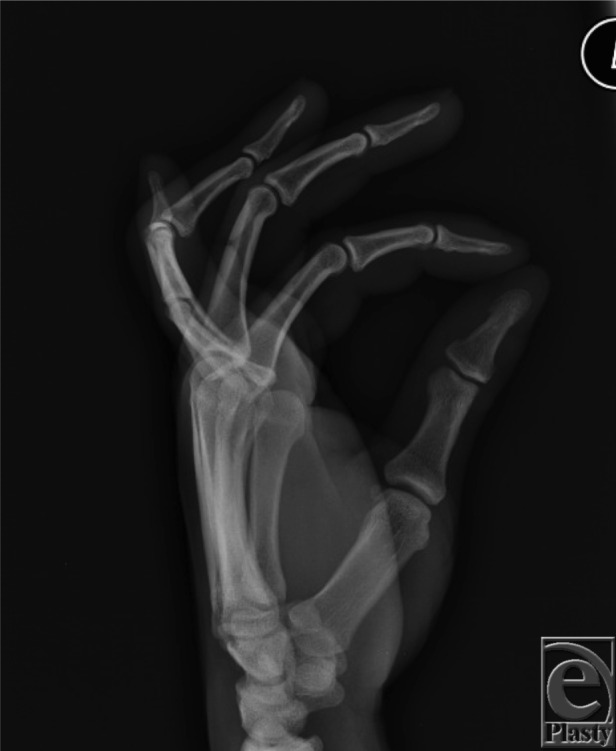
Radiograph demonstrating a *boutonnière* deformity (late) with the proximal interphalangeal joint in flexion and the distal interphalangeal joint in hyperextension.

A 24-year-old man, left-hand-dominant, presented following fall on an outstretched left hand. He had no neurovascular deficits upon examination, with radiographs demonstrating a volar dislocation of his left ring finger proximal interphalangeal joint (Fig 1). The injury was reduced and splinted in extension (Fig 2). Three months after successful reduction and splinting treatment, the patient presented with a *boutonnière* deformity of the affected digit (Fig 3).

## QUESTIONS

1. **What is the mechanism of injury in proximal interphalangeal joint (PIPJ) dislocations and which structures are damaged?**
2. **What are potential late deformities associated with PIPJ dislocation?**
3. **How may late deformities be prevented?**
4. **What are the treatments of these aforementioned late deformities?**

## DISCUSSION

PIPJ dislocations are common hand injuries, with generally good prognoses.^1^ However, despite adequate management, long-term complications may develop. These deformities may be related to the nature of the original injury and the associated soft tissue or bone damage. PIPJ dislocations are classified as dorsal, volar, or lateral depending on the position of the middle phalanx relative to the proximal phalanx 2. Volar dislocations involve injury to the central slip of the extensor tendon with or without disruption of the collateral ligaments and central slip attachment fracture. Dorsal dislocations occur most commonly and feature volar plate disruption with possible volar lip fracture and collateral ligament injury. Lateral dislocations are associated with tears of the collateral ligament complex and possible condylar fracture of the head of the proximal phalanx. 3

Late sequelae of PIPJ dislocations can cause significant disability requiring further management. A chronic *boutonnière* deformity may develop, especially with disruption of the central slip of the extensor tendon.^1^^,^^4^^-^6 Such injury allows the conjoint lateral bands to sublux volarly with flexion, which progresses to a PIPJ extension lag. If untreated, a fixed flexion deformity develops with the lateral bands fixed volarly. The injured central slip allows the lumbrical and interosseous muscles to pull the lateral bands proximally, increasing tension on the terminal tendon and hyperextending the distal interphalangeal joint (DIPJ). In all cases, even with proper care, stiffness and flexion contracture are the most common concerns, and some mobility impairment may persist.^6^^,^^7^ An additional finding may be chronic swelling, which slowly diminishes but never fully resolves,^6^ often due to scar tissue accumulation.^7^ Damage to volar structures, including the volar plate or volar capsule, may cause PIPJ hyperextension, resulting in swan neck deformity, especially with inadequate treatment. In addition, serious concerns are redislocation and subluxation.^6^

Recognition of the type and extent of injury is of paramount importance when preventing later deformity because different dislocations require specific treatment protocols.^2^ Careful history and examination, assessing the mechanism of injury, tenderness, and range of motion in combination with provocative maneuvers and radiographic tests are necessary for diagnosis.^1^ Prevention of a *boutonnière* deformity begins with the initial recognition of central slip injury and splinting of the PIPJ in full extension for 3 weeks.^7^ If central slip disruption is not acknowledged and the finger is splinted with slight PIPJ flexion, development of *boutonnière* deformity is likely.^7^ Stiffness and flexion contracture may be avoided by encouraging early, supervised, active range of motion; however, assessment of joint stability and avoidance of overly aggressive movement are necessary to prevent redislocation and subluxation.^6^ Compression with an elastic dressing manages edema and chronic swelling, which further restrict movement.^6^ Finally, prevention of a swan neck deformity requires immediate recognition of PIPJ hyperextension and treatment with a functional splint that limits extension (ie, figure-of-eight splint).^5^

Should late deformities arise, treatment options are available. A *boutonnière* deformity may be addressed nonsurgically or surgically. Serial casting or dynamic splinting over 6 to 12 weeks may achieve full, passive extension in the affected PIPJ. Active DIPJ flexion exercises, which pull the lateral bands dorsally, are essential. Surgical management may involve pinning the PIPJ in extension with or without central slip repair or a tendon rebalancing procedure. 5 Stiffness can be addressed with serial casting, static or dynamic splinting, or operative release. Chronic swelling can only be managed with compression wrapping, and patients should be counseled accordingly. Fixed swan neck deformities may require open release or PIPJ arthrodesis. Treatment of redislocation is accomplished by addressing the cause (ie, fixation failure, inadequate extension block, excessive movement) and often requires surgical revision or salvage.^6^

PIPJ dislocations are relatively common injuries that, despite generally good prognoses, require adequate attention and care. Late deformities are more likely to develop with inappropriate management and cause significant disability. In cases of volar dislocations, recognition of central slip injury at the time of presentation and extension splinting prevent *boutonnière* deformity. Treatment of *boutonnière* deformity also relies on extension splinting, but operative repair is sometimes necessary.
